# Comparison of Pigment Production by Filamentous Fungal Strains under Submerged (SmF) and Surface Adhesion Fermentation (SAF)

**DOI:** 10.3390/jof9010048

**Published:** 2022-12-28

**Authors:** Liliana R. Rengifo, Paola Rosas, Nicolás Méndez, Yvette Ludeña, Susana Sirvas, Ilanit Samolski, Gretty K. Villena

**Affiliations:** Laboratorio de Micología y Biotecnología “Marcel Gutiérrez—Correa”, Universidad Nacional Agraria La Molina, Lima 15024, Peru

**Keywords:** fungal natural pigments, Submerged Fermentation (SmF), Biofilm Fermentation (BF), Solid-State Fermentation (SSF)

## Abstract

Although synthetic colorants are widely used in many industries due to their high stability at different conditions in industrial processes, evidence of its negative impact on health and the environment is undeniable. Filamentous fungi are well known for their use as alternative sources to produce natural pigments. However, an adequate comparison of the productivity parameters between the fermentation systems could be limited to their heterogeneous conditions. Even though Solid-State Fermentations (SSF) on natural substrates are widely used for pigments production, complex media, and non-controlled variables (T, pH, medium composition), these systems could not only hamper the finding of accurate productivity parameters, but also mathematical modeling and genomics-based optimization. In this context, the present study screened five pigment-producing fungi by comparing Submerged (SmF) and Surface Adhesion Fermentation [biofilm (BF) and Solid-State (SSF)] with defined media and controlled variables. For this purpose, we used the same defined media with sucrose as the carbon source for pigment production on SmF, BF, and SSF, and BF and SSF were carried out on inert supports. Five molecularly identified *Penicillium* and *Talaromyces* strains isolated from the Peruvian rainforest were selected for their ability to produce yellowish-orange colorants. Highest productivities were obtained from *T. brunneus* LMB-HP43 in SmF (0.18 AU/L/h) and SSF (0.17 AU/L/h), and *P. mallochii* LMB-HP37 in SSF (0.18 AU/L/h). Both strains also exhibited the highest yields (AU/g biomass) in the three fermentation systems, reaching values greater than 18-folds in SSF compared to the other strains. Conversely, *T. wortmannii* LMB-HP14 and *P. maximae* LMB-HP33 showed no ability to produce pigments in the SSF system. The performed experiments accurately compared the effect of the fermentation system on yield and productivity. From this, further genomics approaches can be considered for an extensive analysis of pigment synthesis pathways and a genomics-driven optimization in the best fermentation system.

## 1. Introduction

Increasing demand for natural colorants, with an annual growth rate increase of 3.57% and a projected market of about 3.5 billion dollars by 2028, requires continuous prospecting for new and better biological sources, as well as efficient production systems. Main advantages in their use, in contrast to synthetic ones, are related to health and environmental safety. Wide applications of natural colorants include food, cosmetic textiles, and pharmaceutical industries [1,2,3].

Plants and microorganisms are the main source of natural colorants [4,5]. However, colorants produced by plants are seasonal and mostly unstable for light, heat, and pH changes, while extraction methods and yields could limit their commercial applications [6,7]. On the other hand, the production of colorants by microorganisms offers advantages related to having no seasonal dependence, low costs, feasibility for scaling up, and higher yields, among others [5,8,9,10].

Particularly, fungi are known as producers of a wide range of pigments that include carotenoids, melanins, flavins, phenazines, quinones, and, more specifically, monascins and violacein, or indigo [8,11,12,13,14]. Fungal genera such as *Aspergillus*, *Fusarium*, *Penicillium, Monascus*, *Trichoderma*, and *Laetiporus* produce those and many other pigments used to obtain colors such as red, purple, yellow, brown, orange, and green with wide industrial applications [15,16,17].

Regarding production, Submerged Fermentation (SmF) is preferred due to its simplicity in terms of control and suitability for scaling-up; although solid substrates are considered more adequate for fungal pigment production since they provide support for mycelium growth [16]. Pigment production in SmF and Solid-State Fermentation (SSF) is generally based on complex media formulated from diverse heterogeneous substrates, mainly agro-industrial wastes [18,19]. Therefore, carrying out a balancing of the process is a complicated task as yields cannot be attributed to any variable.

A major category called Surface Adhesion Fermentation (SAF), proposed by Gutierrez-Correa and Villena [20], includes Biofilm (BF) and Solid-State Fermentations (SSF). While BF microbial cells initially attach to an inert support submerged in a liquid medium where they grow, forming a biofilm. SSF microorganisms grow on moist solid supports (natural or inert) in the absence of free-flowing water. Both fermentation systems resemble the natural mode of fungal growth, mainly attached to surfaces. In this sense, adhesion-dependent signaling has a pivotal role in productivity, rather than water limitation [20]. For operating conditions, system homogeneity of SAF makes the mathematical modeling and comparison of productivity parameters easier for further optimization and scale-up [21].

In this context, the present work compared pigment production under Submerged (SmF) and Surface Adhesion Fermentation [Biofilm (BF) and Solid-State (SSF)] with defined medium and controlled variables for pigment production as a rational screening design for filamentous fungi isolated from the Peruvian rainforest.

## 2. Materials and Methods

### 2.1. Fungal Strains Selection

Filamentous fungal strains, previously isolated from soils of the undisturbed Macuya Forest (Pucallpa, Peru) [22] and maintained in the culture collection of the Laboratory of Mycology and Biotechnology (Universidad Nacional Agraria La Molina, Lima, Peru), were used in this study. Fourteen strains were screened for pigment production on Potato Dextrose Agar (PDA) plates and Potato Dextrose Broth (PDB) flasks agitated at 180 rpm and incubated at 28 °C for 7 days. Pigment-producing strains were selected and maintained at 4 °C.

### 2.2. Fungal DNA Extraction, Amplification, and Sequencing

Fungal biomass was obtained from liquid cultures and ground with liquid nitrogen using a mortar and pestle based on the procedure reported by Vega et al. (2015) [23]. Subsequently, pulverized biomass was used for DNA extraction following the method described by Möller et al. (1992) [24]. Primers used for amplification of the Internal Transcribed Spacer (ITS), β-tubulin (*BenA*) gene and calmodulin gene (*CaM*), were selected according to Visagie et al. (2014) [25]. PCR amplifications were performed in 20 µL reactions using GoTaq^®^ DNA Polymerase (Promega, Madison, WI, US), following the manufacturer’s protocol and the cycling conditions recommended by the authors. PCR products were visualized on 1% agarose gels. Purification and sequencing of the amplicons was conducted by Macrogen Inc. (Seoul, Korea).

### 2.3. Fungal Identification and Phylogenetic Analysis

DNA sequences were read and manually edited with the BioEdit program v. 7.2.5. (Thomas A. Hall, Raleigh, NC, US). Assembly was performed using the CAP3 program and the sequences obtained were compared to the NCBI GenBank database sequences using the BLASTN tool. Identified sequences were aligned using the Clustal Omega v.1.2.4. program (Desmond Higgins, Fabian Sievers, David Dineen & Andreas Wilm, Dublin, IR) and used to build phylogenetic trees with the MEGA-X program v.10.2.6. (Sudhir Kumar, Koichiro Tamura, Glen Stecher & Michael Li, PA, USA) through the maximum likelihood estimation (MLE) method with 1000 bootstraps.

### 2.4. Inoculum Preparation

For inoculum preparation, each strain was grown in PDA flasks and incubated at 28 °C for 7 days. Then, spores were recovered with 10 mL of 0.1% (*v*/*v*) Tween 80 solution, diluted to a concentration of 10^7^ spores/mL and used as inoculum for SmF and BF. For SSF, a 0.42 × 10^7^ spores/mL solution was used to maintain the final concentration of the inoculum.

### 2.5. Production Medium Composition

Modified Czapek-Dox broth (2 g/L NaNO_3_, 1 g/L K_2_HPO_4_, 0.5 g/L KCl, 0.5 g/L MgSO_4_·7H_2_O, 0.001 g/L FeSO_4_·7H_2_O, 20 g/L sucrose, pH 6.5) was used as production medium in the three fermentation systems. 

### 2.6. Fermentation Systems

Performed fermentation systems included Submerged Fermentation (SmF) and Surface Adhesion Fermentation (SAF) with two variants: Biofilms (BF) and Solid-State Fermentation (SSF). All experiments were performed in triplicate. 

For SmF, 250 mL flasks containing 70 mL of production medium were inoculated with 2.1 mL of spore suspension (3% *v*/*v*) and incubated at 28 °C for up to 10 days in a shaking bath at 175 rpm.

For BF, biofilms were formed on polyester supports following the procedure described by Villena and Gutiérrez-Correa [26], with some modifications. Briefly, flasks containing a pre-weighed 3.1 × 3.1 cm (0.27 cm^2^/mL) piece of polyester cloth and 70 mL of distilled water were used. Each flask was inoculated with 2.1 mL of spore suspension (3% *v*/*v*) and incubated at 28 °C for 15 min at 175 rpm to allow for the spores’ attachment. Then, the unbound spores were washed twice with sterile distilled water. Finally, polyester cloths were transferred to 250 mL sterile flasks containing 70 mL of production medium and incubated in the same conditions for up to 10 days. 

SSF was carried out based on the method described by Gamarra et al. [21] with some modifications. Briefly, horticulture pearls with diameters of 0.7–1.5 mm were used as support. Pearls were previously washed three times with distilled water and dried at 70 °C for 24 h. Then, the apparent density of the pearls was calculated to determine the quantity to be added to each 250 mL flask. The pearls were imbibed with 10 mL of concentrated (7×) production medium, frozen for 2 days, and dried at 60 °C to allow adsorption of the fermentation medium by the pearls. Once the pearls were dried out, the flasks were autoclaved. Finally, each flask was inoculated with 5 mL of spore suspension and distilled water was added to the flasks to maintain 75% moisture within the system. The flasks were then placed inside a moisture chamber and incubated at 28 °C for up to 10 days. 

### 2.7. Extracellular Pigment and Biomass Recovery

Extracellular pigments from SmF and BF were separated from the biomass by vacuum filtration. The filtrate was collected in 50 mL sterile centrifuge tubes and stored for further analysis. Harvested fungal biomass was washed three times with distilled water and dried for weight determination. In the case of BF, non-adhered biomass was separated from the biofilms prior to weight determination.

For SSF, 70 mL of sterile distilled water was added to the flasks and agitated at 180 rpm for 15 min, prior to vacuum filtration. Then, collected supernatants were filtered through syringe filters to remove remnants of pearls and spores, collected in 50 mL sterile centrifuge tubes, and stored for further analysis. In this case, fungal biomass was indirectly determined by quantifying intracellular protein, following the alkaline hydrolysis method described by Sehnem et al. [27], with some modifications. For this purpose, retentates (biomass + support) were washed several times with sterile distilled water and dried at 70 °C for 24 h. Then, different amounts of the dried retentate were weighed and placed in 70 mL glass tubes with screwing caps and 20 mL of 0.2 M NaOH was added to each tube. The tubes were autoclaved at 121 °C for 10 min, and the reaction was neutralized by adding 20 mL of 0.2 M HCl to each tube. After vacuum filtration, intracellular protein concentration was determined from filtrates collected at 550 nm by Lowry’s colorimetric method using the Folin–Ciocalteu reagent with bovine serum albumin (BSA) as the protein standard [28]. Finally, a standard curve was elaborated in order to correlate intracellular protein (mg/mL) with dry biomass (g).

### 2.8. Determination of Pigment Intensities (AU)

The intensity of extracellular pigments was determined by using a UV-visible diode array spectrophotometer (200–800 nm). Once the equipment was calibrated with the corresponding production medium, maximum wavelength peak absorbance (Abs _λmax_) was determined for each fermentation system. Pigment Absorbance Units (AU) were calculated by multiplying the Abs _λmax_ by the dilution factor.

## 3. Results and Discussion

### 3.1. Molecular Identification and Phylogenetic Analysis of Pigment-Producing Fungal Strains

**Figure 1:**
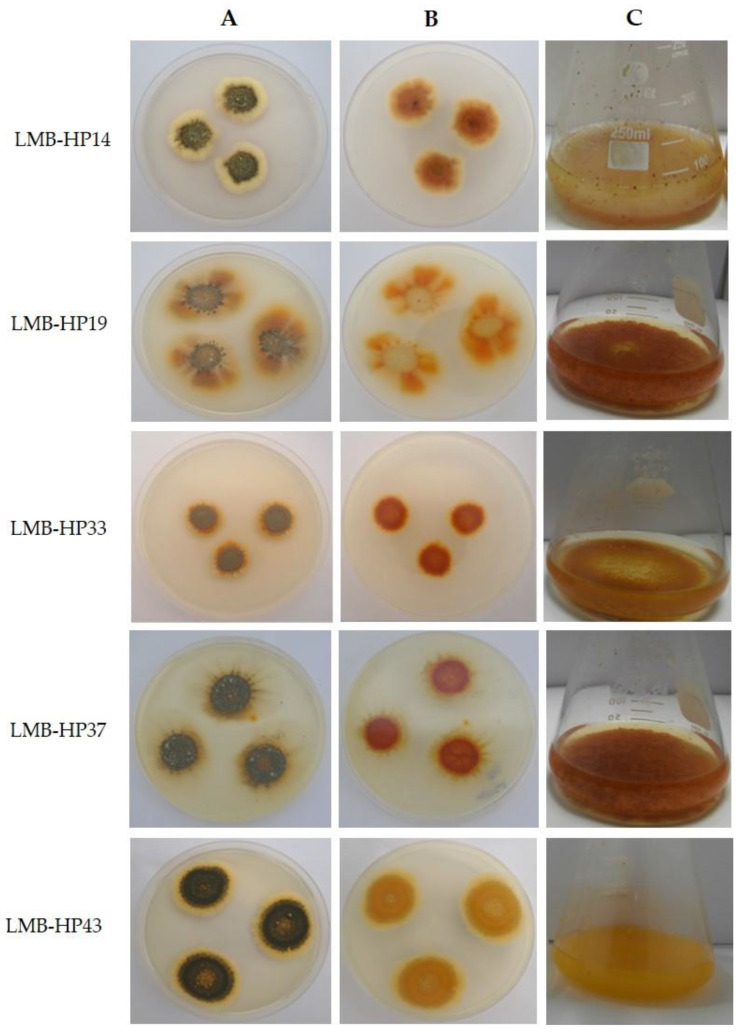
Selected pigment-producing strains after 7 days at 28 °C. Potato Dextrose Agar (PDA) plates showing the obverse (**A**) and reverse (**B**) of fungal colonies. (**C**) Liquid cultures with Potato Dextrose Broth (PDB).

**Figure 2:**
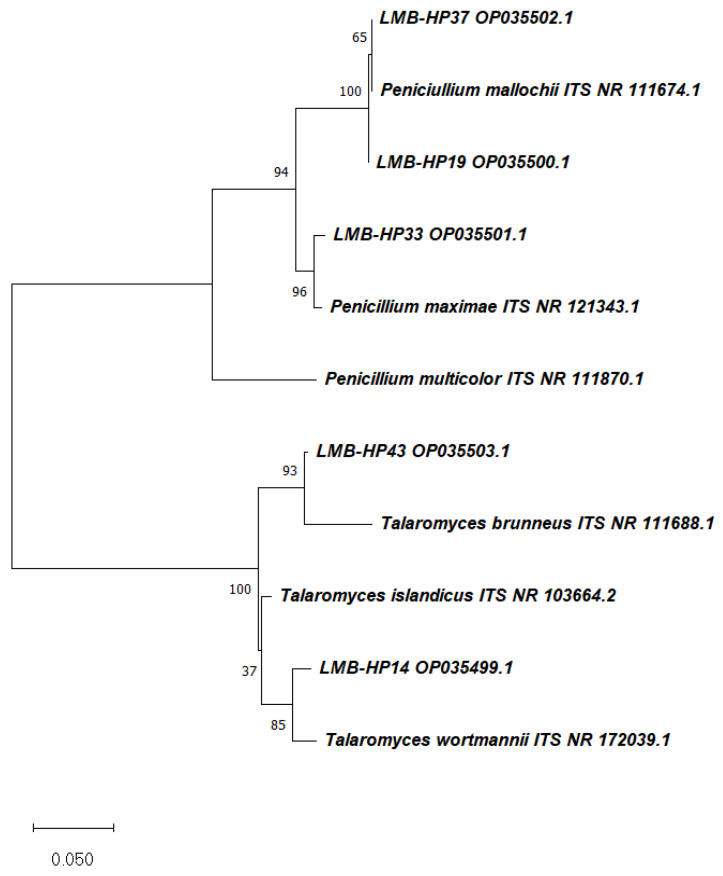
Representative maximum likelihood (ML) phylogenetic tree using Internal Transcribed Spacer (ITS) region of selected pigment-producing strains. The numbers next to the branches indicate the percentage of trees which have the associated taxa clustered together. The scale bar indicates the number of substitutions per site.

Five strains were able to produce yellowish-orange pigments on PDA plates and in PDB flasks under the assayed conditions (Figure 1). Selected fungal strains were molecularly identified as members of the genera *Penicillium* and *Talaromyces*, as shown in Table 1. The representative maximum likelihood (ML) phylogenetic tree obtained for ITS regions is presented in Figure 2.

Several species of *Penicillium* and *Talaromyces* have been reported for their ability to synthetize secondary metabolites, including pigments for biotechnological applications [18,29]. Indeed, the species identified in this study have been taxonomically described, pointing out their characteristic yellowish/orange mycelia [25,30,31,32]. Therefore, studies on species from these genera are currently being focused on due to their potential as cell factories for the safe production of non-toxigenic and stable pigments at the industrial scale [16,18]. However, at present, there have been no studies that accurately compare pigment production systems using the same variables.

Many pigments produced by ascomycetes are polyketides derivatives, mainly the anthraquinones and azaphilones responsible for the yellow and red color produced by many species of *Penicillium* and *Talaromyces* [17,33,34,35,36,37,38]. As secondary metabolites, these polyketide-based pigments exhibit a wide range of biological activities, such as antimicrobial, antifungal, antiviral, antioxidant, cytotoxic, nematicidal, anti-inflammatory, and anti-tumoral activities, among other useful therapeutic applications [39,40,41,42]. However, considering that some *Penicillium* and *Talaromyces* species, including *T. wortmanni*, are known to produce different mycotoxins [43,44], and that some can be co-produced along with pigments, it is necessary to perform a safety evaluation during the bioprocess before being used at the industrial scale, as well as the structural identification of the products [12]. For example, the potential of *Talaromyces purpurogenus* to produce pigments at the industrial scale has been discouraged due to the risk of mycotoxin production; *Talaromyces atroroseus* is able to produce pigments without any co-production of mycotoxins, thus, this species is highly recommended for industrial purposes [45]. 

Recently, an azaphilone pigment rich in sclerotiorin [46] has been obtained from *Penicillium mallochii*, as well as an orange-red pigment [47], whose bioactive effects have important applications in the food industry and medicine. Regarding pigments produced by *Talaromyces brunneus* and *T. wortmannii*, anthraquinone compounds as biemodin and rugulosin with antibiotic effects have been reported [31,48]. To our knowledge, no studies related to pigments produced by *Penicillium maximae* have yet been conducted. Considering that some pigments produced by species of *Talaromyces* are not stable enough for industrial processes [45], an analysis of their stability should be performed.

### 3.2. Pigments Spectra of Selected Fungal Strains

**Figure 3:**
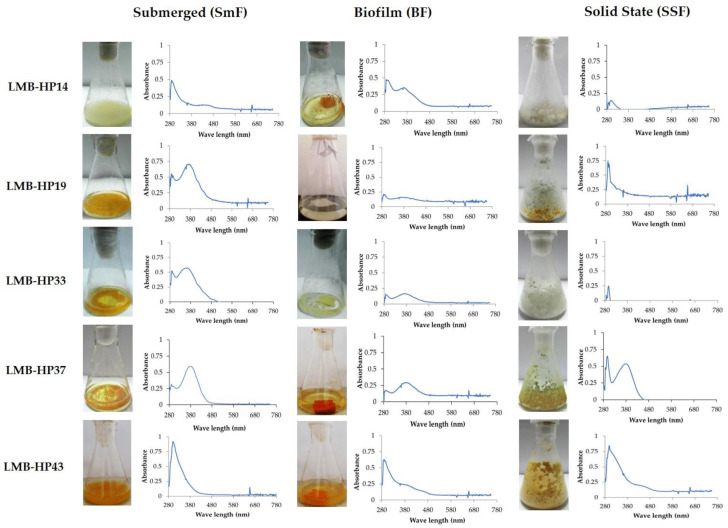
Absorption spectra at UV and visible regions of extracellular pigments of *T. wortmannii* LMB-HP14, *P. mallochii* LMB-HP19, *P. maximae* LMB-HP33, *P. mallochii* LMB-HP37, and *T. brunneus* LMB-HP43 after 7 days in Submerged Fermentation (SmF), Biofilm fermentation (BF), or Solid Satate Fermentation (SSF) systems using production medium.

As shown in Figure 3, after 7 days in the SmF system, maximum absorbance peaks were reached between 360–380 nm, except for pigments secreted by strains *T. wortmannii* LMB-HP14 and *T. brunneus* LMB-HP43, which showed maximum absorbance peaks near 300 nm. The same pattern was observed for pigments secreted in BF, except for *P. mallochii* LMB-HP19 and *P. maximae* LMB-HP33, which did not show any significant peak; whereas, in SSF, the maximum peaks were observed near 300 nm. In the same way, other authors [49,50] have reported species of *Penicillium* and *Talaromyces* with maximum absorption wavelengths in the UV region, although yellow and orange pigments are usually exhibited at 410 and 470 nm, respectively [51]. Accordingly, *Talaromyces albobiverticillius*, *Talaromyces amestolkiae*, and *T. purpureogenus* have been reported to produce yellow-orange pigments in the visible region, although these species are also able to produce a red pigment [37,51,52,53,54,55]. Commonly, chromophores of polyketide-based pigments, such as azaphilones and anthraquinones, absorb in the UV region (200–300 nm), whereas the nature and the number of substituted functional groups determines absorbance in the visible region (400–700 nm) [37]. 

### 3.3. Pigments Productivity and Yields

**Figure 4:**
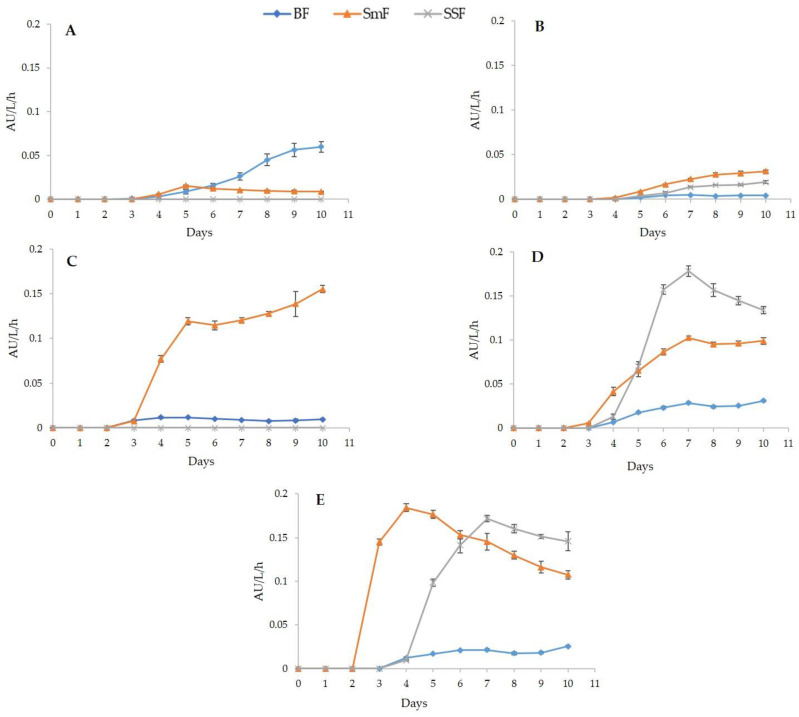
Pigments productivity kinetic curves (AU/L/h) of selected fungal strains cultured in Submerged Fermentation (SmF), Biofilm Fermentation (BF), and Solid State Fermentation (SSF) systems using production medium. (**A**): *T. wortmannii* LMB-HP14, (**B**): *P. mallochii* LMB-HP19; (**C**): *P. maximae* LMB-HP33; (**D**): *P. mallochii* LMB-HP37; (**E**): *T. brunneus* LMB-HP43. Data represent the mean of three replicates.

The capacity to produce pigments by the five selected strains was evaluated during 10 days under SmF and SAF systems (BF and SSF) (Figure 4). It was observed that the highest productivities were obtained from *T. brunneus* LMB-HP43 in both SmF (0.18 AU/L/h) and SSF (0.17 AU/L/h) at 4 and 7 days, respectively, and from *P. mallochii* LMB-HP37 in SSF at 7 days (0.18 AU/L/h); whereas *T. wortmannii* LMB-HP14 and *P. maximae* LMB-HP33 were not able to produce any pigments in SSF. The lowest productivity was observed in BF by all tested strains, except for *T. wortmannii* LMB-HP14, which showed its best performance under this system. Interestingly, for most strains, the pigments were intracellularly retained in SmF and biofilms (see Figure 3). In the same way, other *Penicillium*/*Talaromyces* strains reach the maximum pigment productivity after 5–7 days under SmF, considering that production of secondary metabolites begins in the stationary phase, unlike other strains that undergo a starvation state after several weeks to start producing these metabolites [37,44,56,57].

Pigment yields (AU/g biomass) were also compared among selected strains after 7 days of fermentation under SmF, BF, and SSF systems (Table 2). Although it has already been reported that pigment production may vary according to the fermentation system, its heterogeneity has hampered the comparison of productivity parameters [18]. In contrast, our results have allowed an adequate comparison.

In SmF, *T. brunneus* LMB-HP43 showed the highest yield (1.54 AU/g). The yields obtained under this system were higher than those under BF, except for *T. wortmannii* LMB-HP14. In accordance with SAF advantages, *P. mallochii* LMB-HP37 and *T. brunneus* LMB-HP43 showed the best production yields on SSF system (9.05 and 14.57 AU/g biomass, respectively). Unlike other studies with *Penicillum* and *Talaromyces*, the experimental design used in this study allowed a comparison of pigment production under the same operation variables. Although *Penicillium*, *Talaromyces*, and other genera have been studied for pigment production, it has been mainly carried out on natural substrates [18,19]. Therefore, the substrate composition may be variable and could make difficult the standardization, optimization, and scale-up processes.

It is worth mentioning that, at present, there is not a consensus on the units that should be used to report results, which makes it difficult to compare yields among different pigment production strategies. 

No direct correlation between biomass formation and pigment production has yet been described for *Penicillium* or other fungal species, since they are considered secondary metabolites. Although the pigment production level has been related to genetic and environmental factors [58,59], it was not considered the adhesion influence. That could explain the inverse correlation found between the biomass yield and extracellular pigments (AU) obtained from *Penicillium* and *Talaromyces* species under SSF compared to SmF, and corroborates that SSF significantly favors pigment production by this species [50]. However, optimization is still necessary to improve productivity [18]. In agreement with this, as reviewed by other authors [60], the enhanced yields obtained in SSF might be due to the attachment of the mycelium to the solid surface, a condition that resembles natural fungal growth, rather than to the extractive effect of the solid matrix. Most microorganisms, especially filamentous fungi and many actinomycetes, primarily live and grow in nature under conditions that resemble SSF [20,61], and more than 98% of isolates from marine environments have been obtained from the underwater surfaces of solid substrates [62], as it is the case for pigment-producing strains (*T. albobiverticillius* and *Penicillium* species) obtained from marine sediment, dead coral, and marine plants (endophytes) [37,63,64]. 

Despite the fact that SmF is generally preferred due to its advantages for scaling-up and industrial production, SSF constitutes a better choice in terms of productivity and yield of fungal pigments [56,60,63,65,66]. In the case of *Penicillium sclerotiorum*, pigment production is limited to SSF or to static SmF (liquid-surface cultures). However, production through stirred SmF can be achieved by reducing the pH below 6.0 [56]. 

Nevertheless, the use of natural substrates such as agro-industrial wastes could be used for media formulation in SmF with promising results. A low-cost medium with corn hydrolysate was formulated for pigment production with *T. atroroseus* by co-utilizing xylose and glucose from corn, but biomass growth presented an extended lag phase [67]. Furthermore, a red pigment with antioxidant activity was produced by using *Cicer arietinum* husks as the fermentation substrate for *T. purpureogenus* [53]. Interestingly, a fermentation system that combines a liquid phase with an air phase, where immobilized cells are alternatively submerged and exposed to air, may considerably improve the production of some pigments of *T. purpurogenus* by combining the advantages of SmF and SSF [55]. However, production levels reached by *T. purpurogenus* were not greater than those reached in our study by *P. mallochii* LMB-HP37 and *T. brunneus* LMB-HP43 under SSF.

BF cultures, belonging to the SAF system, also combine the advantages of SmF and SSF, leading to higher productivity. We have previously demonstrated that fungal biofilms are able to produce greater amounts of hydrolytic enzymes, such as cellulases, compared with SmF. Such an increase could be triggered by adhesion through contact between fungal cells and the surface of the support [26,68,69,70]. However, in this study, BF culture mostly accumulates intracellular pigment. Hence, it seems that the low pigment levels obtained from *T. wortmannii* LMB-HP14, *P. mallochii* LMB-HP37, and *T. brunneus* LMB-HP43 under BF were due to pigment retention by the fungal biofilm (see Figure 3). 

For SSF, as indicated by Dufossé (2019) [45], moisture content strongly influences the regulation of pigment synthesis with *Monascus* on complex substrates, such as rice. At high moisture content levels, enzymatic activity of hydrolytic enzymes, such as glucoamylases, increases and pigment synthesis is inhibited because of catabolic repression exerted by the liberated glucose from the substrate. In fact, it has been described that carbon and nitrogen supplementation have an inhibitory effect on fungal pigment production in this fermentation system [71]. Indeed, a comparative transcriptomic approach confirmed that carbon starvation stress contributes to high pigment yields by suppressing central carbon metabolism and increasing the acetyl-CoA pool [45]. 

Moreover, as reported for *Monascus* pigments on natural substrates, the suitability of SSF has also been attributed to the derepression of pigment synthesis due to the diffusion of intracellular pigments from mycelium to the surrounding solid matrix; whereas, in SmF, pigments remain in the mycelium due to their low solubility in the acidified medium, a situation that could favor their degradation by intracellular enzymes [45,72]. To circumvent product inhibition in SmF by the high density of intracellular pigment, a perstractive fermentation could be considered, which would enable pigment solubilization by exportation using nonionic surfactants, while facilitating downstream processing of the product [72,73,74,75]. The use of inert support for SSF could avoid that inhibition mechanism.

Another important factor is related to fungal morphology. It is worth mentioning that pellet formation was observed in SmF for the three tested *Penicillium* strains (Figure 3), and that this type of growth can promote the production of yellow pigments [58,59]. This could explain to some extent the pigment production exhibited by *Penicillium* strains compared to *T. wortmannii* LMB-HP14, which showed a filamentous growth with no pigment formation. On the contrary, despite showing the same growth pattern, *T. brunneus* LMB-HP43 did produce pigments under SmF. It has also been demonstrated that pH has an important impact on pigment production by *Penicillium* and *Talaromyces* under SmF, enhancing its production at neutro-alkaline conditions when natural substrates are used [50]. 

As expected, in accordance with SAF advantages, SSF has enormous potential for industrial pigment production using *P. mallochii* LMB-HP37 and *T. brunneus* LMB-HP43 strains. Moreover, this controlled fermentations system (SAF) could be applied for basidiomycetes, which constitutes another important group for pigment biosynthesis [76] and production, as was reported for *Laetiporus sulphureus* [77] and *Inonotus hispidus* [78], among others. From these results, further approaches including transcriptomic analysis of pigment biosynthesis and genomics-driven optimization processes should be considered, so that productivity parameters can be also validated on natural substrates. Additionally, co-cultures under SSF could be considered a promising strategy by which to increase pigment production yields [79].

## 4. Conclusions

Surface adhesion fermentation, including biofilms (BF) and SSF systems, has been used for pigment production and compared with SmF as a screening strategy for strains of *Penicillium* and *Talaromyces* species isolated from the tropical forest. The main characteristics of the fermentation systems were the use of defined media and inert supports for BF and SSF, so it was possible to control operating variables and medium composition, and accurately compare productivity parameters among all the cultures performed. Our results showed that both *P. mallochii* LMB-HP37 and *T. brunneus* LMB-HP43 are promising strains for natural pigment production on SSF conditions, which agree with the advantages of SAF systems. Nevertheless, further genomics approaches are needed to dilucidate pigment biosynthesis mechanisms, as well as to perform rational optimization strategies.

## Figures and Tables

**Table 1:** Fungal identities of selected pigment-producing strains.

Fungal Strain	Identity	Reference	Accession N°
LMB-HP14	*Talaromyces wortmannii*	This work	OP035499
LMB-HP19	*Penicillium mallochii*	This work	OP035500
LMB-HP33	*Penicillium maximae*	This work	OP035501
LMB-HP37	*Penicillium mallochii*	Vega et al. [21]	OP035502
LMB-HP43	*Talaromyces brunneus*	This work	OP035503

**Table 2:** Pigment production yields (AU/g biomass) of *T. wortmannii* LMB-HP14, *P. mallochii* LMB-HP19, *P. maximae* LMB-HP33, *P. mallochii* LMB-HP37, and *T. brunneus* LMB-HP43, cultured during 7 days in Submerged Fermentation (SmF), Biofilm Fermentation (BF), and Solid State Fermentation (SSF) systems using production medium.

Strain	Yield (AU/g)		
	Fermentation Systems		
		Surface Adhesion Fermentation (SAF)	
	Submerged (SmF)	Biofilm (BF)	Solid-State (SSF) *
LMB-HP14	0.05 ± 0.01	0.64 ± 0.02	0.00 ± 0.00
LMB-HP19	0.20 ± 0.01	0.13 ± 0.01	0.78 ± 0.03
LMB-HP33	0.29 ± 0.01	0.18 ± 0.03	0.00 ± 0.00
LMB-HP37	0.62 ± 0.03	0.43 ± 0.03	9.05 ± 0.34
LMB-HP43	1.54 ± 0.07	0.62 ± 0.14	14.57 ± 0.33

(*) Biomass was calculated from intracellular protein. Data represent the mean of three replicates.
